# Added value of dedicated axillary hybrid 18F-FDG PET/MRI for improved axillary nodal staging in clinically node-positive breast cancer patients: a feasibility study

**DOI:** 10.1007/s00259-017-3823-0

**Published:** 2017-09-14

**Authors:** Thiemo J. A. van Nijnatten, B. Goorts, S. Vöö, M. de Boer, L. F. S. Kooreman, E. M. Heuts, J. E. Wildberger, F. M. Mottaghy, M. B. I. Lobbes, M. L. Smidt

**Affiliations:** 1 Department of Radiology and Nuclear Medicine, Maastricht University Medical Center+, P.O. Box 5800, 6202 AZ Maastricht, The Netherlands; 2 Department of Surgery, Maastricht University Medical Center+, Maastricht, The Netherlands; 3 GROW – School for Oncology and Developmental Biology, Maastricht University Medical Center+, Maastricht, The Netherlands; 4 Division of Internal Medicine, Department of Medical Oncology, Maastricht University Medical Center+, Maastricht, The Netherlands; 5 Department of Pathology, Maastricht University Medical Center+, Maastricht, The Netherlands; 6 Department of Nuclear Medicine, University Hospital, RWTH Aachen University, Aachen, Germany

**Keywords:** Breast cancer, Lymph node imaging, Hybrid PET/MRI, Axilla

## Abstract

**Purpose:**

To investigate the feasibility and potential added value of dedicated axillary 18F-FDG hybrid PET/MRI, compared to standard imaging modalities (i.e. ultrasound [US], MRI and PET/CT), for axillary nodal staging in clinically node-positive breast cancer.

**Methods:**

Twelve patients with clinically node-positive breast cancer underwent axillary US and dedicated axillary hybrid 18F-FDG PET/MRI. Nine of the 12 patients also underwent whole-body PET/CT. Maximum standardized uptake values (SUVmax) were measured for the primary breast tumor and the most FDG-avid axillary lymph node. A positive axillary lymph node on dedicated axillary hybrid PET/MRI was defined as a moderate to very intense FDG-avid lymph node. The diagnostic performance of dedicated axillary hybrid PET/MRI was calculated by comparing quantitative and its qualitative measurements to results of axillary US, MRI and PET/CT. The number of suspicious axillary lymph nodes was subdivided as follows: N0 (0 nodes), N1 (1–3 nodes), N2 (4–9 nodes) and N3 (≥ 10 nodes).

**Results:**

According to dedicated axillary hybrid PET/MRI findings, seven patients were diagnosed with N1, four with N2 and one with N3. With regard to mean SUVmax, there was no significant difference in the primary tumor (9.0 [±5.0] vs. 8.6 [±5.7], *p* = 0.678) or the most FDG-avid axillary lymph node (7.8 [±5.3] vs. 7.7 [±4.3], *p* = 0.767) between dedicated axillary PET/MRI and PET/CT. Compared to standard imaging modalities, dedicated axillary hybrid PET/MRI resulted in changes in nodal status as follows: 40% compared to US, 75% compared to T2-weighted MRI, 40% compared to contrast-enhanced MRI, and 22% compared to PET/CT.

**Conclusions:**

Adding dedicated axillary 18F-FDG hybrid PET/MRI to diagnostic work-up may improve the diagnostic performance of axillary nodal staging in clinically node-positive breast cancer patients.

## Introduction

Breast cancer is one of the most prevalent types of invasive cancer among women [1]. The involvement of axillary lymph nodes is an important prognostic factor, as 5-year overall survival is reduced from 98% to 85% in the case of lymph node metastases [2]. Therefore, European guidelines recommend preoperative axillary imaging to differentiate between patients with and without clinically node-positive breast cancer [3]. Accurate lymph node staging is important, as it affects the type of axillary surgery and indications for systemic therapy and radiotherapy fields [4].

In addition to differentiating between node-negative and node-positive disease, an equally important consideration is the number of suspicious nodes. Current treatment plans are based on the number of suspicious lymph nodes (i.e. one to three versus four or more) according to imaging findings and histopathological confirmation prior to the start of therapy [3]. Preoperative axillary imaging currently involves the use of axillary ultrasound (US), with tissue sampling in the case of suspicious nodal findings. Schipper et al. demonstrated that more than 50% of patients with one to three suspicious lymph nodes on US had four or more lymph node metastases at surgery [5]. This percentage decreased to 11–15% when standard breast MRI was used for axillary nodal staging [6]. In addition, the use of a dedicated axillary MR protocol in place of a standard breast MR protocol further improves the diagnostic performance of MRI for nodal staging [7]. Research suggests that PET/CT has the highest accuracy for preoperative nodal staging: Koolen et al. demonstrated that 98% of all patients with suspicious axillary lymph nodes on PET/CT would eventually have axillary lymph node metastases after surgery [8].

Hybrid PET/MRI allows for the simultaneous use of the promising axillary nodal staging techniques PET and MRI [9]. Therefore, the aim of this study was to investigate the feasibility and potential added value of dedicated axillary 18F-FDG hybrid PET/MRI compared to standard imaging modalities (i.e. axillary US, MRI and PET/CT) for axillary nodal staging in patients with clinically node-positive breast cancer.

## Material and methods

### Setting and patients

After approval of the study by the local medical ethical committee, a total of 12 patients provided signed informed consent to undergo dedicated axillary hybrid PET/MRI. Inclusion criteria were patients with histopathologically confirmed clinically node-positive breast cancer. Exclusion criteria were contraindications for either MRI, including the contrast agent gadobutrol, or PET, including radiotracer 18F–fluorodeoxyglucose (18F-FDG). Prior to inclusion, patients underwent axillary US. In addition, all patients were scheduled for MR imaging and, depending on the number of suspicious lymph nodes (i.e. 1–3 vs. 4+), for PET/CT as well [10].

### Axillary ultrasound

Axillary US exams were performed by the radiologist on call, using an ultrasound system with a linear 2- to 17-MHz array transducer (iU22-xMATRIX, Philips Healthcare, Best, the Netherlands). Criteria for suspicious axillary lymph nodes included diffuse cortical thickening, focal cortical mass and/or thickening, and loss of fatty hilum [11]. During axillary US, tissue sampling of suspicious axillary lymph nodes was performed. In the case of multiple suspicious nodes, only the most suspicious node was sampled, and the number of suspicious axillary lymph nodes was reported.

### PET/CT

In the Netherlands, PET/CT exams can be considered in the case of stage III/IV breast cancer [10, 12]. After patients had fasted for a period of at least 4 h and had a blood glucose level < 10 mmol/L, an intravenous injection of 18F-FDG was administered at a dose of 2 MBq/kg body weight, followed by a resting period of 60 ± 10 min. Patients were placed in supine position with elevated arms in the PET/CT scanner (Gemini TF, Philips Healthcare, Best, the Netherlands). A low-dose CT scan (120 kV, 30 mAs, slice thickness 4 mm) from head to thigh was performed, followed by the PET acquisition (3 min per bed position). CT images were reconstructed using filtered back-projection. PET images were reconstructed using the BLOB-OS-TF time-of-flight algorithm provided by the manufacturer, with a voxel size of 4 × 4 × 4 mm^3^.

### Hybrid PET/MRI

Directly following whole-body PET/CT, state-of-the-art dedicated axillary hybrid PET/MRI images were acquired on a 3.0 T Biograph mMR integrated PET/MRI system (Siemens Healthineers, Erlangen, Germany). Dedicated axillary MRI was performed using a six-channel anterior body coil (Siemens Healthineers), which was placed on the ipsilateral shoulder. Patients were placed in prone position with both arms elevated.

In three cases, no whole-body PET/CT was performed. These patients underwent dedicated axillary hybrid PET/MRI after administration of 18F-FDG at a dose of 2 MBq/kg body weight and a resting period of 60 ± 10 min, with identical fasting time and a blood glucose level < 10 mmol/L as with PET/CT.

The dedicated axillary hybrid PET/MRI protocol consisted of an unenhanced T2-weighted (T2W) sequence, contrast-enhanced T1-weighted sequence (TW1), and a fusion sequence of PET images with the unenhanced T2W sequence. All sequences were performed in coronal view.

More specifically, a 2D standard T2W sequence without fat suppression was performed first using the following scan parameters: repetition time (TR) 2000 ms; echo time (TE) 118 ms; variable degree flip angle; turbo spin echo (TSE) factor 41; averages 1.4; voxel size 1.1 × 1.1 × 1.1 mm; field of view (FOV) 220 × 220 mm^2^; matrix 192 × 192, resulting in an in-plane resolution of 1.15 × 1.15 mm^2^. Next, gadobutrol (Gadovist^®^, Bayer HealthCare, Berlin, Germany) contrast at a volume of 15 cm^3^ was administered intravenously. After 6 min, a 3D T1W sequence was acquired using the following parameters: TR 7.27 ms; TE 2.46 ms; 15° flip angle; averages 2; voxel size 1.1 × 1.1 × 1.1 mm; FOV 220 × 220 mm^2^; matrix 192 × 192; resulting in an in-plane resolution of 1.15 × 1.15 mm^2^. Total acquisition time of the MRI exam was 20 min, 21 s.

Three-dimensional iterative reconstruction was performed for all PET images of the hybrid PET/MRI system (number of iterations 3; subsets 21; voxel size 4.1 × 2.6 × 3.9 mm^3^; image matrix 172 × 172; zoom 1.0), with automatic attenuation correction through implementation of a four-compartment model attenuation map (Dixon-based μ-map). Images were acquired in a single bed position within 15 min after the start of MRI acquisition on the hybrid PET/MRI system.

### Image evaluation

All axillary lymph nodes on dedicated axillary MR, hybrid PET/MRI and PET/CT images were evaluated by a breast radiologist (M.B.I.L.) and nuclear medicine physician (S.V.). The breast radiologist has 7 years of dedicated experience in breast imaging, and the nuclear medicine physician has 9 years of experience in nuclear imaging.

First, the radiologist scored each axillary lymph node on dedicated axillary T2W images, according to the criteria previously defined by Baltzer er al., using a confidence scale from 0 (no lymph nodes) to 4 (definitely malignant) [13]. Lymph nodes with a score of 0–2 were considered benign, and those with a score of 3–4, malignant. Suspicious characteristics included irregular margins, inhomogeneous cortex, perifocal edema, absence of fatty hilum, asymmetry, and absence of chemical shift artifacts [13–15]. Next, contrast-enhanced dedicated axillary T1W images were shown, which allowed the radiologist to adjust his score for each lymph node, if deemed necessary. Suspicious characteristics included absence of contrast hyperintensity of the lymph node and absence of an intact nodal border [15].

Second, the breast radiologist and nuclear medicine physician evaluated in consensus each axillary lymph node on dedicated axillary hybrid PET/MRI using a four-point system as previously described by Aukema et al. for the assessment of lymph nodes with 18F-FDG PET/CT: 0 = similar to other surrounding lymph nodes, 1 = slightly more intense than other lymph nodes, 2 = moderately intense, 3 = very intense [16]. Lymph nodes with a score of 0–1 were considered benign, while a score of 2–3 was considered malignant. PET information was considered to be decisive regarding the number of suspicious axillary lymph nodes in the evaluation of dedicated axillary hybrid PET/MRI. The nuclear medicine physician further measured maximum standardized uptake values (SUVmax) of the primary breast tumor and the most FDG-avid axillary lymph node on each dedicated axillary hybrid PET/MRI exam. Finally, the nuclear medicine physician evaluated each axillary lymph node on 18F-FDG PET/CT, using the same four-point system [16], and measured SUVmax of the primary breast tumor and the most FDG-avid axillary lymph node on each PET/CT exam. Both PET/CT and PET/MRI exams were shown anonymously and in randomized order to the nuclear physician for evaluation of the number of suspicious axillary lymph nodes.

### Pathology

In this study, clinically node-positive disease was based on US-guided cytological and/or histological confirmation of axillary lymph node biopsy. In cases where US was performed in the referring hospital, the pathology of the axillary lymph node biopsy was revised and metastases were verified by the pathologist in our hospital (L.K.).

### Statistical analyses

Statistical analyses were performed using SPSS software (version 22, IBM Corp., Armonk, NY, USA). Axillary nodal status was based on the number of suspicious axillary lymph nodes, as follows: none (N0), 1–3 (N1), 4–9 (N2), or 10 or more (N3), according to axillary US (including results of tissue sampling), dedicated axillary (PET)MRI and/or PET/CT, respectively. When the result of dedicated axillary hybrid PET/MRI led to either up- or down-staging of clinical nodal status compared to standard imaging modalities (US, MRI or PET/CT), it was considered a change in clinical nodal status according to dedicated axillary hybrid PET/MRI findings.

In patients who underwent both PET/CT and hybrid PET/MRI, the Wilcoxon signed-rank test was used to compare the mean SUVmax of the primary tumor (± standard deviation [SD]) and the most FDG-avid axillary lymph node (± SD) on PET/CT with hybrid PET/MRI. *P*-values (two-sided) < 0.05 were considered statistically significant.

## Results

### Patients

A total of 12 consecutive patients with clinically node-positive breast cancer over a period from April 2016 to March 2017 were included in this feasibility study. The mean age was 49 years (range, 28–70 years). In all patients, clinically positive nodal status was histopathologically confirmed with US-guided biopsy. Eleven of 12 patients had invasive carcinoma of no special type; one patient had mixed lobular carcinoma with carcinoma of no special type. In addition, 9 of 12 patients underwent PET/CT. A more detailed overview of the general characteristics is shown in Table 1.

**Table 1:** General patient characteristics

Mean age (years) (range)	49 (28–70)
Mean clinical tumor size (mm) (range)	46 (13–150)
cT-stage (%)	
cT1	2 (17%)
cT2	4 (33%)
cT3	3 (25%)
cT4	3 (25%)
Multifocal tumor (%)	
No	8 (67%)
Yes	4 (33%)
Tumor type (%)	
Invasive carcinoma NST	11 (92%)
Mixed NST and lobular	1 (8%)
Tumor grade (%)	
2	6 (50%)
3	6 (50%)
Hormone receptor/HER2 status	
ER/PR-positive, HER2-negative	4 (33%)
ER/PR-negative, HER2-positive	1 (8%)
Triple-negative	2 (17%)
ER/PR-positive, HER2-positive	5 (42%)

*Abbreviations: cT-stage* clinical tumor stage, *NST* no special type, *ER* estrogen, *PR* progesterone, *HER2* human epidermal growth factor receptor 2

### Nodal staging

When compared to standard imaging modalities, dedicated axillary hybrid PET/MRI resulted in changes to clinical nodal status as follows: 40% according to US findings (4 of 10 patients), 75% according to T2W MRI findings (9 of 12 patients), 40% according to CE-T1W MRI findings (4 of 10 patients) and 22% according to PET/CT findings (2 of 9 patients), respectively. Differences between PET/CT and PET/MRI findings were due to better delineation of the lymph nodes on the MRI component. A more detailed overview of all cases, including up- or down-staging of nodal status according to dedicated axillary hybrid PET/MRI findings, is provided in Table 2.

**Table 2:** Clinical nodal status (including number of suspicious lymph nodes) according to US, PET/CT, T2W MRI, CE-T1W MRI and hybrid PET/MRI

Patient	US	PET/CT	Dedicated axillary (PET/)MRI		
			T2W MRI	CE-T1W MRI	Hybrid PET/MRI
1	N2 (4)	N2 (5)	N3 (10) ↓	N3 (10) ↓	N2 (8)
2	N1 (1)	–	N0 ↑	N1 (1)	N1 (2)
3	N1 (1)	–	N2 (5) ↓	N2 (7) ↓	N1 (1)
4	N1 (3) ↑	N2 (5)	N3 (10) ↓	N2 (9)	N2 (8)
5	N+ (−)	N2 (9)	N1 (3) ↑	–	N2 (7)
6	N2 (6) ↓	N1 (2)	N3 (25) ↓	N3 (11) ↓	N1 (2)
7	N+ (−)	N2 (4) ↓	N2 (4) ↓	–	N1 (3)
8	N1 (1)	N1 (3)	N2 (6) ↓	N2 (7) ↓	N1 (3)
9	N2 (5) ↑	N2 (7) ↑	N3 (10)	N3 (13)	N3 (10)
10	N1 (1)	N1 (1)	N1 (2)	N1 (2)	N1 (1)
11	N1 (2) ↑	–	N1 (3) ↑	N2 (5)	N2 (4)
12	N1 (1)	N1 (1)	N1 (2)	N1 (3)	N1 (1)

*Abbreviations: PET* positron emission tomography, *CT* computed tomography, *MRI* magnetic resonance imaging, *T2W* T2-weighted, *CE-T1W* contrast-enhanced T1-weighted, *N0* no suspicious lymph nodes, *N1* 1–3 suspicious lymph nodes, *N2* 4–9 suspicious lymph nodes, *N3* 10 or more suspicious lymph nodes, *N+* clinically node-positive status, *−* missing, *↓* down-staging according to result of hybrid PET/MRI, *↑* up-staging according to result of hybrid PET/MRI

In two patients, axillary US findings regarding the number of suspicious axillary lymph nodes was missing, because US was performed in the referring hospital. However, in addition, neither patient underwent contrast-enhanced T1W MRI, as this was refused by both patients.

**Fig. 1:**
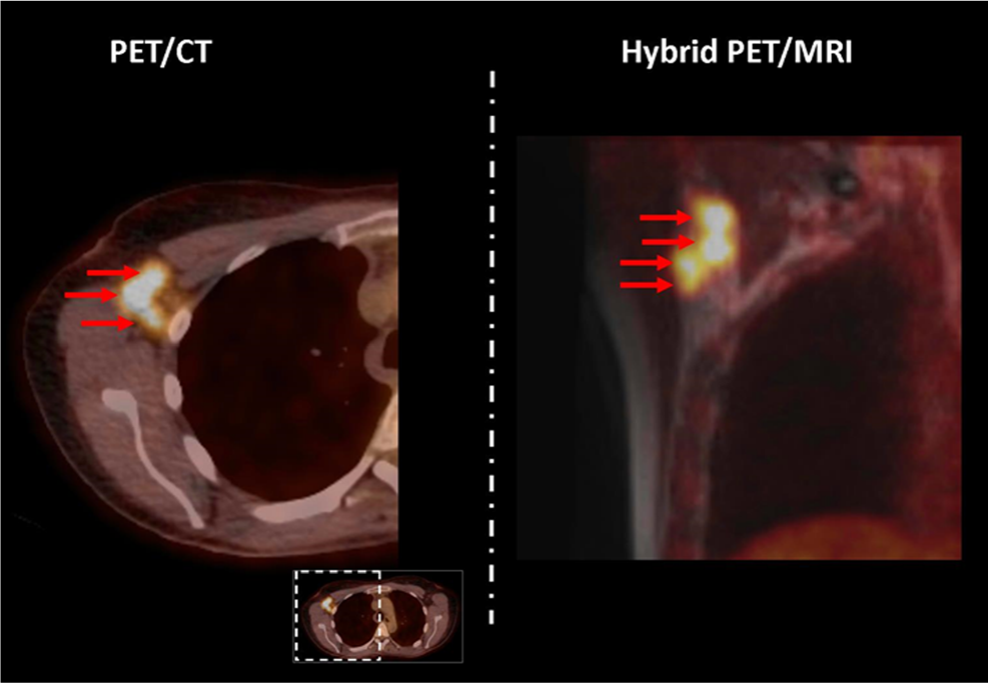
Example of a patient with a 35-mm invasive carcinoma of no special type in her right breast. One cross-sectional PET/CT image (*left*) of the right axilla demonstrates three suspicious lymph nodes (*red arrows*) out of a total of five suspicious lymph nodes on PET/CT. One coronal dedicated axillary hybrid PET/MRI image (*right*) demonstrates four suspicious lymph nodes (*red arrows*), with a total of eight suspicious lymph nodes on dedicated axillary hybrid PET/MRI. The most dorsally located suspicious lymph node on (low-dose) PET/CT consists of two suspicious lymph nodes on dedicated axillary hybrid PET/MRI

**Fig. 2:**
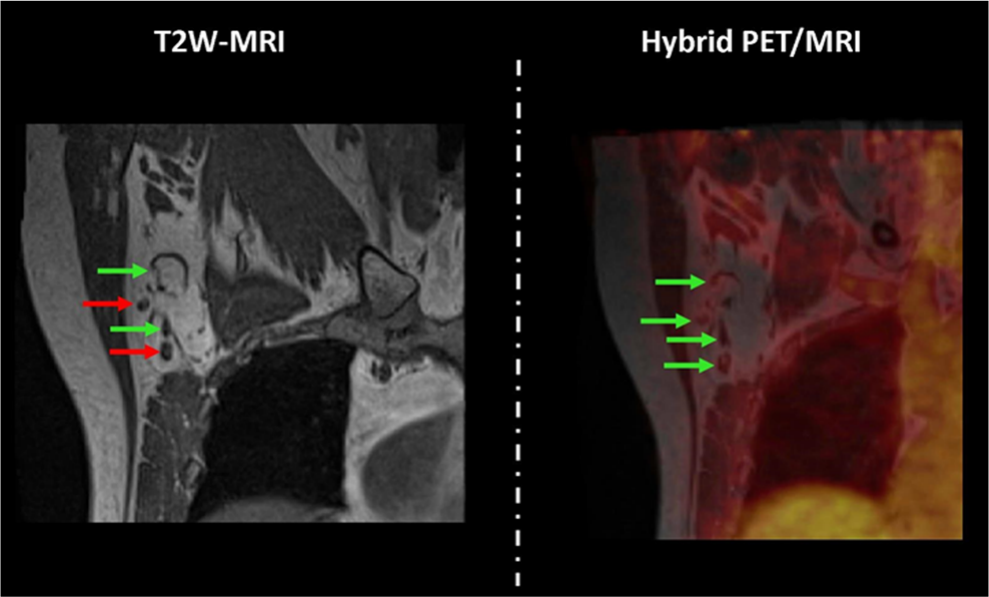
Example of a patient with a 31-mm invasive carcinoma of no special type in her right breast. A coronal T2-weighted MR image (*left*) of the right axilla demonstrates two (*red arrows*) of four suspicious nodes. A coronal dedicated axillary hybrid PET/MRI image (*right*) demonstrates no FDG-uptake in any of the four nodes (*green arrows*), which resulted in four negative nodes on dedicated axillary hybrid PET/MRI

Figures 1 and 2 illustrate examples of differences in the number of suspicious axillary lymph nodes between PET/CT (Fig. 1) and T2W MRI (Fig. 2) when compared to dedicated axillary hybrid PET/MRI. The Appendix provides a complete overview of all axillary lymph nodes evaluated on dedicated axillary (PET/)MRI and PET/CT images.

### SUVmax measurements

The mean SUVmax of the primary tumor and the most FDG-avid axillary lymph node on PET/CT were 8.6 (± 5.7) and 7.7 (± 4.3), respectively. Comparable values were achieved on hybrid PET/MRI, with mean SUVmax of 9.0 (± 5.0; *p* = 0.678) and 7.8 (± 5.3; *p* = 0.767) for the primary tumor and the most FDG-avid lymph node, respectively. Table 3 provides an overview of all SUVmax measurements on PET/CT and hybrid PET/MRI for each patient.

**Table 3:** Per-patient overview of SUVmax measurements on PET/CT and hybrid PET/MRI

Patient	PET/CT SUVmax	Hybrid PET/MRI SUVmax
1		
Primary tumor	9.3	12.0
Axillary lymph node	8.7	11.0
2		
Primary tumor	11.1	11.7
Axillary lymph node	16.0	20.1
3		
Primary tumor	8.8	7.6
Axillary lymph node	7.7	4.6
4		
Primary tumor	5.1	10.3
Axillary lymph node	2.0	1.7
5		
Primary tumor	19.5	17.1
Axillary lymph node	11.9	8.5
6		
Primary tumor	3.4	3.9
Axillary lymph node	6.6	5.7
7		
Primary tumor	1.8	1.5
Axillary lymph node	4.3	5.4
8		
Primary tumor	4.0	4.5
Axillary lymph node	3.7	5.0
9		
Primary tumor	14.1	12.1
Axillary lymph node	7.9	8.0
Mean		
Primary tumor	8.6	9.0
Axillary lymph node	7.7	7.8

*Abbreviations: PET* positron emission tomography, *CT* computed tomography, *MRI* magnetic resonance imaging, *SUVmax* maximum standard uptake value

## Discussion

The aim of this study was to investigate the feasibility and potential added value of dedicated axillary 18F-FDG hybrid PET/MRI compared to standard imaging modalities (i.e. US, MRI and PET/CT) for axillary nodal staging in patients with clinically node-positive breast cancer. The results of our study show that dedicated axillary 18F-FDG hybrid PET/MRI is clinically feasible, and resulted in a change in nodal status in 40–75% of patients when compared to US or MRI. When compared to PET/CT only, nodal status was changed in two of nine patients (22%), although SUVmax measurements were comparable between the imaging modalities.

The importance of accurate differentiation in clinically node-positive patients—i.e. one to three versus four or more positive nodes—prior to start of treatment is a topic of debate, since adjuvant (radiation) therapy planning is based on pre-treatment axillary imaging findings in the case of neoadjuvant systemic therapy [17]. With regard to patients with limited axillary disease after surgery, the American College of Surgeons Oncology Group (ACOSOG) Z0011 trial demonstrated that there was no prognostic benefit in adjuvant axillary treatment in clinically node-negative patients treated with primary surgery [18]. On the other hand, patients with advanced axillary disease (i.e. 4+ positive nodes) do require adjuvant regional treatment [17, 19]. Consequently, pre-treatment axillary imaging is becoming increasingly important as a means of truly identifying patients with four or more versus three or fewer positive nodes. However, long-term outcome for patients treated with additional therapy after the detection of a larger number of positive nodes—for instance, on PET/CT as opposed to ultrasound—remains unclear.

In contrast to our study, Grueneisen et al. found no differences in PET/MRI findings compared to PET/CT and MRI for axillary nodal staging in breast cancer patients [20]. However, in that study, a (standard) breast PET/MRI protocol was used, unlike the state-of-the-art dedicated axillary PET/MRI protocol in our study. In addition, a previous study demonstrated that in approximately 40% of all patients who underwent standard breast MRI, the field of view of the axillary region was not complete [6]. In contrast to Grueneisen, a study by Melsaether et al. showed improved diagnostic accuracy of PET/MR imaging versus PET/CT for axillary nodal staging in breast cancer patients, with an improvement in sensitivity from 88% to 100% [21]. Nonetheless, their study differentiated only node-negative from node-positive disease, rather than considering the number of positive nodes. The results of our study can thus be considered to provide added clinical value compared to standard imaging modalities (US, MRI and PET/CT), further improving a patient-tailored approach for axillary nodal staging in clinically node-positive breast cancer.

The accuracy of MRI for axillary nodal staging in our study may differ from that in standard clinical practice. We used dedicated (coronal) axillary MRI instead of standard (cross-sectional) breast MRI that is most commonly used in clinical practice, which might have resulted in higher accuracy than with standard breast MRI. A previous systematic review by Kuijs et al. similarly demonstrated superior results for axillary nodal staging when a dedicated axillary MRI protocol was used [7]. However, our study revealed that dedicated axillary hybrid PET/MRI is of added value even compared to dedicated axillary T2W and contrast-enhanced MRI, as it still resulted in changes to clinical nodal status in 75% and 40% of cases, respectively. In addition, differences in the performance of PET/CT can occur when PET/CT is performed with hanging breast acquisition rather than standard PET/CT. Teixeira et al. demonstrated that PET/CT with hanging breast acquisition improved detection of regional lymph node metastases over standard PET/CT [22].

SUVmax measurements of the primary tumor (8.6 vs. 9.0) and the most FDG-avid axillary lymph node (7.7 vs. 7.8) in our study were comparable between PET/CT and hybrid PET/MRI, despite different reconstruction protocols between imaging modalities. Pujara et al. also found a strong correlation in SUVmax between PET/CT and PET/MRI in breast cancer patients, and concluded that SUVs from PET/MRI could be used for quantification of 18F-FDG activity [23]. However, SUVmax comparisons between FDG-avid axillary lymph nodes on PET/CT and PET/MRI were not reported in their study. The results of our study can thus support the earlier findings of Pujara et al. for using SUVmax of FDG-avid axillary nodes on PET/MRI for 18F-FDG activity quantification.

In the future, whole-body PET/MRI, including an integrated dedicated axillary PET/MRI protocol, might replace whole-body PET/CT for locoregional and distant staging in breast cancer, since both locoregional and distant staging seem to be at least as accurate as PET/CT [20, 24–26]. A secondary benefit would be the reduction in radiation exposure to patients of up to 64% compared to PET/CT [21]. The cost-effectiveness of PET/MRI must also be further investigated.

Limitations of the current study include the missing data concerning correlation of dedicated axillary hybrid PET/MR images and final histopathology of axillary lymph nodes; however, nodal metastases were histopathologically confirmed in all patients. Moreover, FDG-avid axillary lymph nodes can be considered malignant, since previous studies have reported high specificity of 95–100% for the detection of axillary lymph node metastases in breast cancer [16, 27, 28]. However, because of the lack of final histopathology in these cases, it is possible that some benign lymph nodes according to FDG-activity on PET/MR still could have been malignant.

Second, there was a timing difference in the performance of PET/MRI in our cohort. Three of 12 patients underwent only PET/MRI, instead of PET/CT following PET/MRI. This might have led to differences in SUVmax measurements on PET/MRI between the two subgroups [12]. These three patients were thus excluded from subgroup analysis regarding SUVmax measurements on PET/MRI and PET/CT.

Third, patients underwent unenhanced low-dose whole-body PET/CT rather than diagnostic contrast-enhanced PET/CT. The results of PET/CT should be interpreted with this important limitation in mind.

Finally, hybrid PET/MRI is currently available in only a few hospitals worldwide. Therefore, other techniques should also be further explored to approximate the diagnostic performance of PET/MRI—for instance, by combining hanging breast PET images with MRI images [29].

In conclusion, this feasibility study demonstrated that, compared to standard imaging modalities, dedicated axillary 18F-FDG hybrid PET/MRI appears to improve diagnostic performance for axillary nodal staging in clinically node-positive breast cancer patients. Further studies are needed to investigate the accuracy of this hybrid modality and to compare its performance with that of other dedicated imaging tools, such as hanging breast PET/CT.

## Appendix

**Table 4:** Complete overview of all axillary lymph nodes evaluated on (PET)/MRI and PET/CT exams

	T2W MRI		CE-T1W MRI		Hybrid PET/MRI		PET/CT	
	Benign (score 0–2)	Suspicious (score 3–4)	Benign (score 0–1)	Suspicious (score 2–3)	Benign (score 0–1)	Suspicious (score 2–3)	Benign (score 0–1)	Suspicious (score 2–3)
Patient								
1	2	10	2	10	4	8	1	5
2	11	0	10	1	9	2	–	–
3	15	5	13	7	19	1	–	–
4	2	10	3	9	4	8	0	5
5	4	3	–	–	0	7	2	9
6	3	25	17	11	26	2	5	2
7	6	4	–	–	7	3	0	4
8	9	6	8	7	12	3	0	3
9	4	10	1	13	4	10	0	7
10	9	2	9	2	10	1	0	1
11*	5	3	5	5	6	4	–	–
12	4	2	3	3	5	1	0	1

*This concerned the patient with mixed lobular carcinoma with carcinoma of no special type

*Abbreviations: T2W* T2-weighted, *MRI* magnetic resonance imaging, *CE-T1W* contrast-enhanced T1-weighted, *PET* positron emission tomography, *CT* computed tomography
